# Supplementary material for: Trajectories of Allopregnanolone and Allopregnanolone to Progesterone Ratio across the Six Subphases of Menstrual Cycle
Source: Biomolecules. 2023 Apr 5;13(4):652. doi: 10.3390/biom13040652 (PMC10135782; doi:10.3390/biom13040652)
Supplement: Supplementary file 1 [file biomolecules-13-00652-s001.zip › biomolecules-2244746-supplementary.pdf]

Supplementary Material

Supplementary Table S1. Algorithm to schedule clinic visits (in days) based on average self-reported cycle length in the BioCycle Study (Mumford et al., 2011)

Average cycle length (days)	Visit 1 Menses (M)	Visit 2 Mid follicular (MF)	Visit 3 Periovulatory 1 (O1)	Visit 4 Periovulatory 2[a] (O2)	Visit 5 Periovulatory 3 (O3)	Visit 6 Early luteal (L1)	Visit 7 Mid luteal (L2)	Visit 8 Late luteal (L3)
21	2	4	5	6	7	11	15	20
22	2	4	6	7	8	12	16	21
23	2	5	7	8	9	13	17	22
24	2	5	8	9	10	14	18	23
25	2	6	9	10	11	15	19	24
26	2	6	10	11	12	16	20	25
27	2	7	11	12	13	17	21	26
28	2	7	12	13	14	18	22	27
29	2	7	13	14	15	19	23	28
30	2	8	14	15	16	20	24	29
31	2	8	15	16	17	21	25	30
32	2	9	16	17	18	22	26	31
33	2	9	17	18	19	23	27	32
34	2	10	18	19	20	24	28	33
35	2	10	19	20	21	25	29	34

[a] Luteinizing hormone surge

Supplementary Table S2. Algorithm for aligning the day of the luteinizing hormone (LH) surge (visit 4) on the standardized LH surge visit (O2) (based on the Biocycle study, Mumford et al., 2011)

Standardized Cycle Phase [b]									
	LH peak occurred on visit (*n*)	Menses (M)	Mid follicular (F1)	Periovulatory 1 (O1)	Periovulatory 2 (LH surge; O2)	Periovulatory 3 (O3)	Early luteal (L1)	Mid luteal (L2)	Late luteal (L3)
Early LH peak	Visit 3 (115) *Periovulatory 1*	Visit 1	Visit 2	*Missing* [a]	Visit 3	*Average* of visits 4 & 5	Visit 6	Visit 7	Visit 8
Correctly timed	Visit 4 (139) *Periovulatory 2 (LH surge)*	Visit 1	Visit 2	Visit 3	Visit 4	Visit 5	Visit 6	Visit 7	Visit 8
Late LH peak	Visit 5 (98) *Periovulatory 3*	Visit 1	Visit 2	*Average* of visits 3 & 4	Visit 5	*Missing*	Visit 6	Visit 7	Visit 8
	Visit 6 (30) *Early luteal phase*	Visit 1	*Average* of visits 3, 4 & 5	*Missing*	Visit 6	*Missing*	Visit 7	Visit 8	*Missing*
	Visit 7 (19) *Mid luteal phase*	Visit 1	*Average* of visits 3, 4 & 5	*Missing*	Visit 7	*Missing*	Visit 8	*Missing*	*Missing*
	Visit 8 (5) *Late luteal phase*	Visit 1	*Average* of visits 3, 4 & 5	*Missing*	Visit 8	*Missing*	*Missing*	*Missing*	*Missing*

[a] 'Missing' indicates that after realignment there was no serum collection (visit) during that phase of the cycle such that the reclassified visit was set to missing.
[b] If we assume a standard 28-day cycle, the standardized cycle phases would correspond to approximately days 2, 7, 12, 13, 14, 18, 22 and 27, respectively. Alternatively, these visits could be referenced from the day of the LH surge of a 28-day cycle as: onset of menses until day -8 relative to the LH surge, day -7 to -2, day -1, day 0, day +1, day +2 to day +7, day +8 to day +11, day +12 to end of cycle.

Supplementary Table S3. Progesterone subphase comparisons

Timepoint Comparison		Class	t-statistic	p-value	Adjusted p-value	Significance
Early Follicular	Mid-Follicular	Imputed	5.085	$2.91x10^{-05}$	$5.45x10^{-05}$	**
Early Follicular	Periovulatory	Imputed	-2.219	0.0390	0.0390	*
Early Follicular	Early Luteal	Imputed	-21.373	$3.49x10^{-18}$	$1.75x10^{-17}$	***
Early Follicular	Mid-Luteal	Imputed	-27.452	$6.85x10^{-20}$	$1.03x10^{-18}$	***
Early Follicular	Late Luteal	Imputed	-6.066	$3.93x10^{-05}$	$6.56x10^{-05}$	**
Mid-Follicular	Periovulatory	Imputed	-4.642	0.0001	0.0002	***
Mid-Follicular	Early Luteal	Imputed	-18.770	$7.09x10^{-17}$	$2.66x10^{-16}$	***
Mid-Follicular	Mid-Luteal	Imputed	-23.467	$8.22x10^{-19}$	$6.17x10^{-18}$	***
Mid-Follicular	Late Luteal	Imputed	-8.275	$2.93x10^{-07}$	$6.28x10^{-07}$	***
Periovulatory	Early Luteal	Imputed	-14.366	$1.82x10^{-10}$	$4.55x10^{-10}$	***
Periovulatory	Mid-Luteal	Imputed	-14.080	$1.27x10^{-11}$	$3.80x10^{-11}$	***
Periovulatory	Late Luteal	Imputed	-3.871	0.0008	0.0009	***
Early Luteal	Mid-Luteal	Imputed	-3.934	0.0005	0.0007	***
Early Luteal	Late Luteal	Imputed	4.148	0.0009	0.0009	***
Mid-Luteal	Late Luteal	Imputed	6.346	$5.80x10^{-05}$	$8.70x10^{-05}$	**
Early Follicular	Mid-Follicular	Realigned	4.473	0.0002	0.0002	***
Early Follicular	Periovulatory	Realigned	-2.064	0.0500	0.0500	*
Early Follicular	Early Luteal	Realigned	-19.349	$3.79x10^{-16}$	$1.90x10^{-15}$	***
Early Follicular	Mid-Luteal	Realigned	-24.843	$1.24x10^{-18}$	$1.86x10^{-17}$	***
Early Follicular	Late Luteal	Realigned	-5.504	$4.81x10^{-05}$	$8.02x10^{-05}$	***
Mid-Follicular	Periovulatory	Realigned	-4.495	0.0002	0.0002	***
Mid-Follicular	Early Luteal	Realigned	-16.106	$2.71x10^{-13}$	$1.02x10^{-12}$	***
Mid-Follicular	Mid-Luteal	Realigned	-21.420	$3.16x10^{-16}$	$1.90x10^{-15}$	***
Mid-Follicular	Late Luteal	Realigned	-8.018	$8.38x10^{-07}$	$1.80x10^{-06}$	***
Periovulatory	Early Luteal	Realigned	-14.932	$5.38x10^{-13}$	$1.61x10^{-12}$	***
Periovulatory	Mid-Luteal	Realigned	-13.813	$1.27x10^{-12}$	$3.18x10^{-12}$	***
Periovulatory	Late Luteal	Realigned	-2.652	0.0180	0.0193	*
Early Luteal	Mid-Luteal	Realigned	-3.287	0.0040	0.0050	**
Early Luteal	Late Luteal	Realigned	3.070	0.0090	0.0104	*
Mid-Luteal	Late Luteal	Realigned	5.943	$2.06x10^{-05}$	$3.86x10^{-05}$	***

* p≤0.05 **p≤0.01 ***p≤0.001
